# Association between implant apex and sinus floor in posterior maxilla dental implantation: A three-dimensional finite element analysis

**DOI:** 10.3892/etm.2015.2205

**Published:** 2015-01-22

**Authors:** XU YAN, XINWEN ZHANG, WEICHAO CHI, HONGJUN AI, LIN WU

**Affiliations:** 1 Department of Prosthodontics, School of Stomatology, China Medical University, Shenyang, Liaoning 110001, P.R. China; 2 Center of Implant Dentistry, School of Stomatology, China Medical University, Shenyang, Liaoning 110001, P.R. China; 3 School of Astronautics, Harbin Institute of Technology, Harbin, Heilongjiang 150001, P.R. China

**Keywords:** finite element analysis, dental implants, stress distribution, resonance frequency, sinus floor, implant apex, cortical bone

## Abstract

The aim of the present study was to evaluate the effect of the association between the implant apex and the sinus floor in posterior maxilla dental implantation by means of three-dimensional (3D) finite element (FE) analysis. Ten 3D FE models of a posterior maxillary region with a sinus membrane and different heights of alveolar ridge with different thicknesses of sinus floor cortical bone were constructed according to anatomical data of the sinus area. Six models were constructed with the same thickness of crestal cortical bone and a 1-mm thick sinus floor cortical bone, but differing heights of alveolar ridge (between 10 and 14 mm). The four models of the second group were similar (11-mm-high alveolar ridge and 1-mm-thick crestal cortical bone) but with a changing thickness of sinus floor cortical bone (between 0.5 and 2.0 mm). The standard implant model based on the Nobel Biocare^®^ implant system was created by computer-aided design (CAD) software and assembled into the models. The materials were assumed to be isotropic and linearly elastic. An inclined force of 129 N was applied. The maximum von Mises stress, stress distribution, implant displacement and resonance frequencies were calculated using CAD software. The von Mises stress was concentrated on the surface of the crestal cortical bone around the implant neck with the exception of that for the bicortical implantation. For immediate loading, when the implant apex broke into or through the sinus cortical bone, the maximum displacements of the implant, particularly at the implant apex, were smaller than those in the other groups. With increasing depth of the implant apex in the sinus floor cortical bone, the maximum displacements decreased and the implant axial resonance frequencies presented a linear upward tendency, but buccolingual resonance frequencies were hardly affected. This FE study on the association between implant apex and sinus floor showed that having the implant apex in contact with, piercing or breaking through the sinus floor cortical bone benefited the implant stability, particularly for immediate loading.

## Introduction

It is well known that there are numerous factors that influence the stability of an implant, such as the amount of bone surrounding the implant and the quality of that bone (1), the size (2) and type (3) of implant and whether it is associated with one or two bony cortices (4). Implant length has proved to be an important factor for the success of implantation, particularly for the atrophied posterior maxilla area (5,6); however, to the best of our knowledge, none of these studies have evaluated the association between implant apex and sinus floor cortical bone. Jeong *et al* (7) reported that bicortical implantation had the potential to increase the initial stability and reduce the stress of the cortical bone around the implant neck.

A key factor for the success or failure of a dental implant is the manner in which stresses are transferred to the surrounding bone (8), particularly for immediate loading implantation. It is not difficult to imagine that the sinus floor cortical bone can provide a support force for the implant. As the implant apex gets closer to the sinus floor, the cortical bone will stop the stress distribution and provide a bigger supporting force for the implant; however this conjecture is not easily verified by clinical studies.

Finite element analysis (FEA) has been widely used to predict the effect of clinical factors on the success of implantation, and also to estimate the biomechanical performance associated with various alveolar bone and dental implant conditions (9). FEA allows the prediction of the stress distribution in the contact area of the implants with cortical bone, and around the apex of the implants in the surrounding bone. This method is advantageous for solving complex structural problems as it divides them into smaller and simpler inter-related sections through the use of mathematical techniques (10). Resonance frequency analysis (RFA) is a nondestructive measurement that has been extensively used in clinical practice over the last five years (11). Resonance frequency, as a type of physical property, can also be simulated by FEA and has already been used in the biomechanical research of dental implantation (12,13).

In the present study, three-dimensional (3D) FE models and an implant were constructed. All factors other than the distance between the implant apex and the sinus floor cortical bone were excluded, in order to observe the association between them.

## Materials and methods

### Implant system

The standard implant with a diameter of 4.0 mm and a length of 10.0 mm was modeled and placed in the 3D FE models of the sinus area. The shape and structure of the implant was modeled according to the Nobel Biocare^®^ implant system (Nobel Biocare, Kloten, Switzerland). In order to simplify the analysis, the implant and the abutment were modeled as a unit.

### Sinus geometric modeling

3D-CAD models of the posterior maxilla with 0.3-mm thick (14) sinus membranes, different heights of alveolar ridge and different thicknesses of sinus floor cortical bone were generated using CAD software (SolidWorks 2012, Fukuoka, Japan). The geometry of the maxilla was defined by a bucco-palatal section according to the anatomical aspects of the sinus area (15–18). Six models (models 1-1 to 1-6) were used to research different distances between the implant apex and sinus floor cortical bone. The alveolar ridge heights of these models were between 10 and 14 mm, with 1 mm crestal cortical bone and sinus floor cortical bone. For model 1-1, the implant apex just broke through the sinus floor cortical bone (the upper surface of the sinus floor cortical bone and the apical surface of the implant were at the same level); for model 1-2, the implant apex broke through half the thickness of the sinus floor cortical bone; for model 1-3, the implant apex just made contact with the lower surface of the sinus floor cortical bone; and for the remaining models the implant apexes gradually deviated from the sinus floor. The other four models (models 2-1 to 2-4), with the same alveolar ridge height of 11 mm, were generated to investigate the different depths that the implant was embedded in different thicknesses of sinus floor cortical bone. The thickness of the sinus floor cortical bone changed from 0.5 mm to 2.0 mm in increments of 0.5 mm; relative to the sinus floor cortical bone the implant apex was thus separate, in contact with, penetrating through one-quarter of the bone thickness or penetrating through one-half of the bone thickness, respectively. According to the design of the study, models 2-2 and 1-3 were the same: Alveolar ridge height, 11 mm; crestal cortical bone thickness, 1 mm; and sinus floor cortical bone thickness, 1 mm.

### Material properties

The material properties of different types of tissue, as well as the titanium implants in the models, were assumed to be homogeneous, isotropic and linearly elastic. Young’s modulus, Poisson’s ratio and the mass density of the materials used in the analysis were taken from the literature (2,19,20) and are shown in Table I.

### Interface conditions

The models were prepared with two types of interface conditions: One represented ideal osseointegration for traditional loading (loaded onto the body without force), with 100% union between the implants and maxilla; for the other type, the implant-bone interface was assumed to be a frictional interface (prior to osseous integration, i.e. immediate loading). In total, there were thus four groups: Groups 1 and 2 were based on models 1-1 to 1-6 with interface conditions of either immediate or conventional loading, respectively; groups 3 and 4 were based on models 2-1 to 2-4, also with interface conditions of either immediate or conventional loading, respectively. To ensure initial stability for the immediate loading condition, the model was constructed using nonlinear frictional contact elements, which allowed minor displacements between the implant and bone. Under these conditions, the contact zone transfers pressure and tangential forces (i.e. friction) but no tension. The friction coefficient between the implant and bone was set to 0.3 (21).

### Loading and boundary conditions

**Figure 1:**
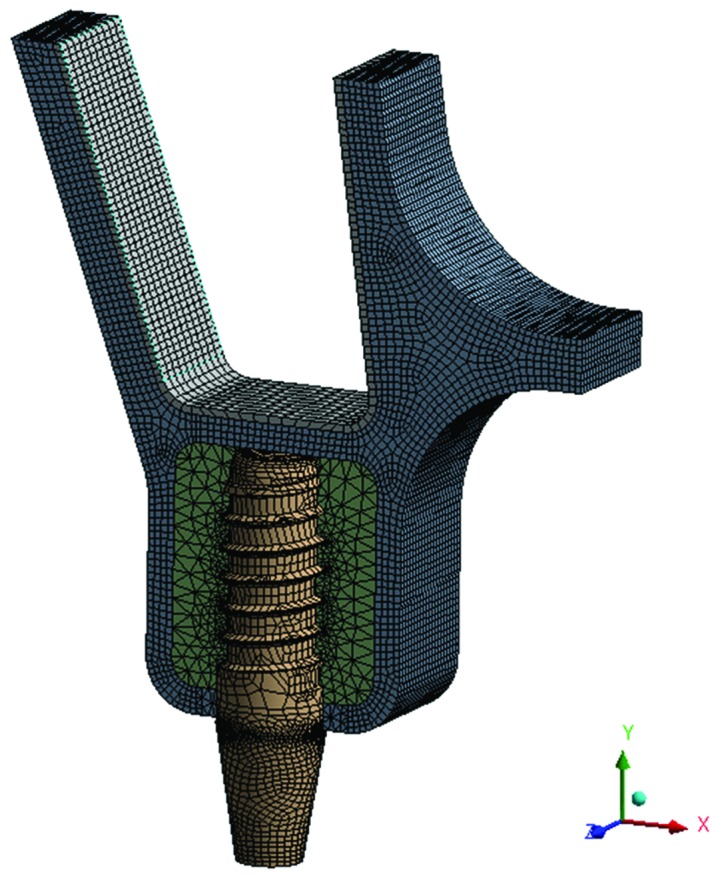
Finite element model of sinus area dental implantation with bone containing 357,801 nodes and 101,050 elements. Sample model 1-3 is shown, exhibiting the following characteristics: Height of alveolar ridge, 11 mm; thickness of crestal cortical bone, 1 mm; sinus floor cortical bone, 1 mm; and a standard implant.

An average force of 129 N (22) inclined 30° posteriorly relative to the implant axis and 30° away from the sagittal plane was dispersed on the top of the implant abutment. ANSYS 12.1 FE software (ANSYS Inc., Harbin, China) was used for the FEA. The models were constrained in all directions at the nodes on the medial and distal bone surfaces, the top of the simulated sinus, the sinus walls and the sinus membrane. The models were meshed with four-node tetrahedral elements and eight-node hexahedral elements and composed of total elements varying from 94,453 to 106,347 and total nodes ranging from 333,087 to 369,874 (Fig. 1). To assess the distribution of stresses, maximum von Mises stresses were visualized with stress contour plots. The biomechanical effects were also analyzed by considering the maximum displacement of the implant neck and apex. Additionally, buccolingual and axial resonance frequencies of the implant were analyzed.

### Statistical analysis

Data were evaluated by t-tests, and P≤0.05 was considered to indicate a statistically significant difference.

## Results

### Stress distribution and maximum von Mises stress

#### Cortical bones of the alveolar ridge and sinus floor

**Figure 2:**
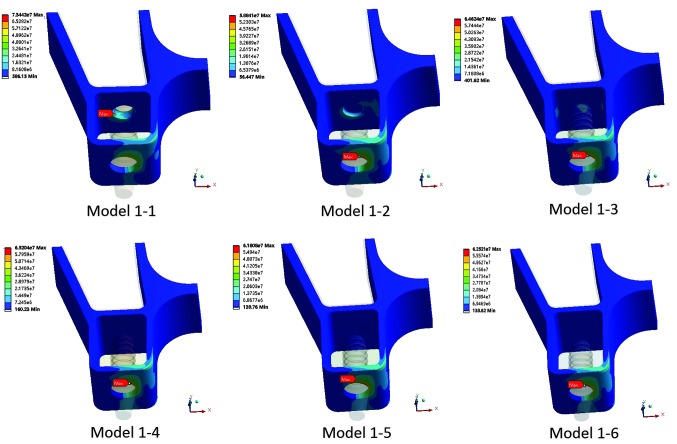
Stress distributions of cortical bones in immediate loading in groups 1 and 2. Maximum von Mises stress was concentrated in the crestal cortical bone around the implant neck with the exception of that in model 1-1.

**Figure 3:**
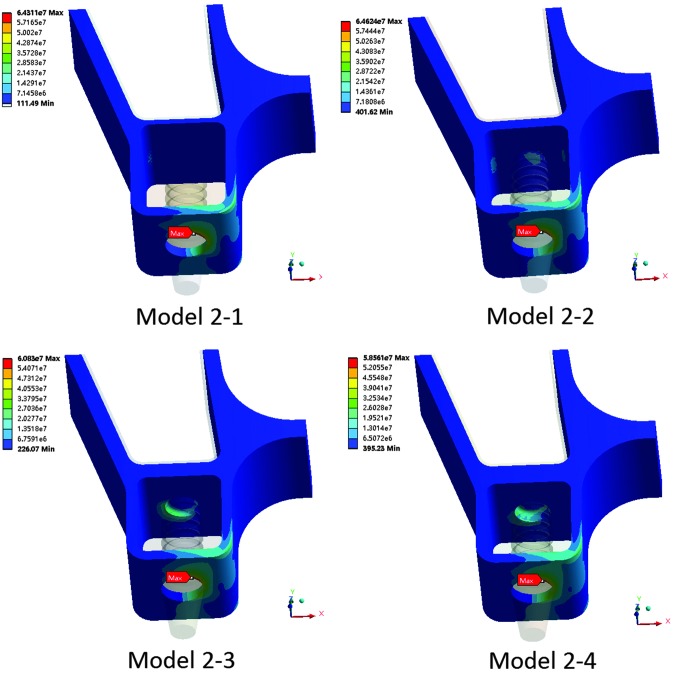
Stress distributions of cortical bones in immediate loading in groups 3 and 4. Maximum von Mises stress of the sinus floor cortical bone increased significantly.

**Figure 4:**
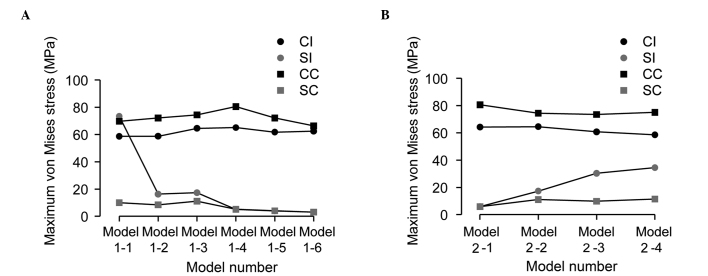
Plot of stress distributions of cortical bones. (A) Groups 1 and 2; (B) groups 3 and 4. Alterations were made in (A) the distance between the implant apex and sinus floor cortical bone and (B) the depth of implant apex breakthrough into the sinus floor cortical bone. CI, crestal cortical bone of immediate loading; SI, sinus floor cortical bone of immediate loading; CC, crestal cortical bone of conventional loading; SC, sinus floor cortical bone of conventional loading.

The stress distributions of the cortical bone in immediate loading are shown in Fig. 2 (groups 1 and 2) and Fig. 3 (groups 3 and 4). Maximum von Mises stresses were also analyzed and are shown in Fig. 4A (groups 1 and 2) and Fig. 4B (groups 3 and 4).

The von Mises stress was concentrated on the surface of the crestal cortical bone around the implant neck, with the exception of that in model 1-1 (immediate loading) (Figs. 2 and 3). In model 1-1 (bicortical implantation), the implant apex broke through the sinus floor cortical bone, which resulted in the sinus floor cortical bone suffering more stress (73.44 MPa) than the crestal cortical bone (58.69 MPa) (Fig. 4A). The results of Fig. 4A show that the maximum von Mises stress of immediate loading was ~18% lower than that of conventional loading. The maximum von Mises stress of the crestal cortical bone increased with the increasing distance between the implant apex and the upper surface of the sinus floor cortical bone. Until the implant apex separated from the lower surface of the sinus floor cortical bone (model 1-4), the stress reached a peak value and then decreased with increasing distance, whether the loading was immediate or conventional. The stress of the sinus floor cortical bone was higher with immediate loading than that with conventional loading prior to the implant apex and sinus floor cortical bone separating. When there was cancellous bone between the implant apex and the sinus floor cortical bone, the stress of the sinus floor cortical bone was approximately the same in immediate and conventional loading.

The results of Fig. 4B show that the different penetration distances of the implant apex into the sinus floor cortical bone had little effect on the maximum von Mises stress of the crestal cortical bone (P>0.05); however the stress of the sinus floor cortical bone with immediate loading was affected, and the maximum von Mises stress increased from 6.01 to 34.48 MPa.

#### Sinus membrane

**Figure 5:**
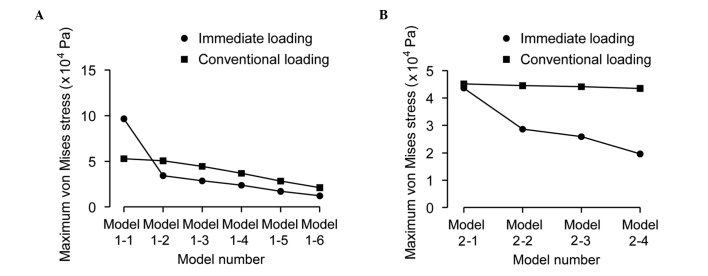
Maximum von Mises stresses of the sinus membranes. (A) Groups 1 and 2 (models 1-1 to 1-6); (B) groups 3 and 4 (models 2-1 to 2-4).

Maximum von Mises stresses of the sinus membrane were analyzed and are shown in Fig. 5A (groups 1 and 2) and Fig. 5B (groups 3 and 4). Fig. 5A shows that, as the distance increased between the implant apex and the upper surface of the sinus floor cortical bone, the maximum von Mises stress decreased significantly, particularly between models 1-1 and 1-2, with immediate loading. Fig. 5B shows that changing the distance that the implant apex pierced into the sinus floor cortical bone had little effect in reducing the maximum von Mises stress of the sinus membrane in conventional loading (P>0.05) but showed a marked effect in immediate loading; in immediate loading, the maximum von Mises stress decreased from 4.36×10^4^ to 1.96×10^4^ Pa.

#### Implant displacement

**Figure 6:**
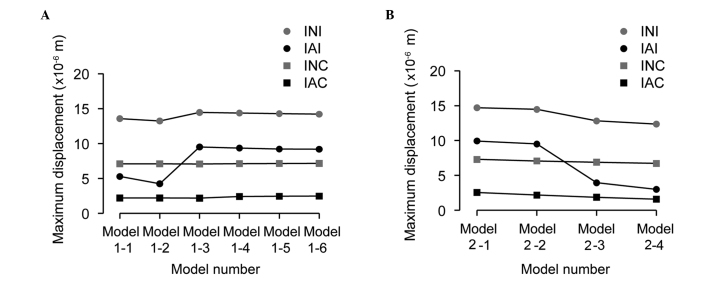
Maximum displacements of the implant. (A) Groups 1 and 2 (models 1-1 to 1-6); (B) groups 3 and 4 (models 2-1 to 2-4). INI, implant neck of immediate loading; IAI, implant apex of immediate loading; INC, implant neck of conventional loading; IAC, implant apex of conventional loading.

**Figure 7:**
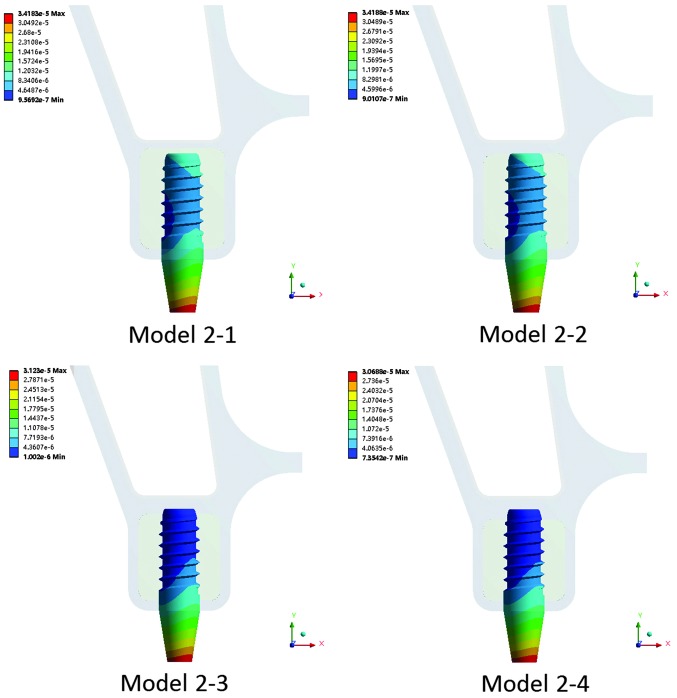
Implant displacement cloud chart of group 3. Maximum displacement decreased with the depth the implant apex pierced the sinus floor cortical bone.

The data of the maximum displacements of the implant neck and apex are shown in Fig. 6A (groups 1 and 2) and Fig. 6B (groups 3 and 4). The implant displacement cloud chart of group 3 is shown in Fig. 7 as an example. The results showed that the maximum displacement of the implant neck was bigger than that of the implant apex in all the models. For immediate loading, when the implant apex broke through or inside the sinus floor cortical bone, the implant maximum displacements, particularly for the implant apex, were smaller than those for the other conditions (Fig. 6A). As the depth the implant apex reached into the sinus floor cortical bone increased, the maximum displacements decreased (Fig. 6B). For conventional loading, as the distance between the implant apex and the sinus floor cortical bone increased, the maximum displacements of the implant neck and apex increased, although inconspicuously. As the depth the implant apex reached into the sinus floor cortical bone increased, the maximum displacements of the implant apex decreased significantly.

#### Implant resonance frequencies

**Figure 8:**
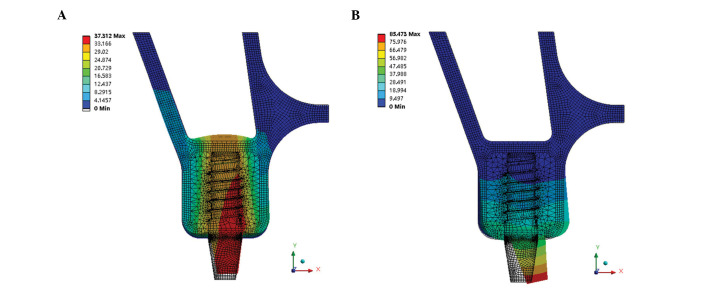
Vibrational modes of the bone-implant complex. (A) Axial mode; (B) bending mode.

**Figure 9:**
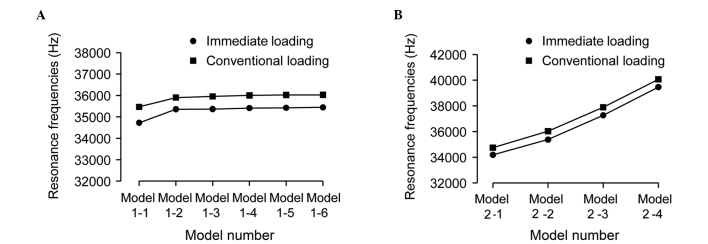
Implant axial resonance frequencies. (A) Groups 1 and 2; (B) groups 3 and 4. Implant axial resonance frequency increased significantly with the depth that the implant apex broke into the sinus floor cortical bone.

**Figure 10:**
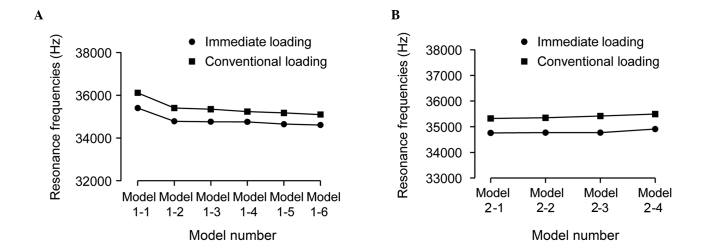
Implant buccolingual resonance frequencies. (A) Groups 1 and 2; (B) groups 3 and 4. Implant buccolingual resonance frequencies showed no clear differences with the bone changes around the implant apex.

Fig. 8 shows the two vibrational modes of the implant-bone system. The data of implant axial resonance frequencies are shown in Fig. 9A (groups 1 and 2) and Fig. 9B (groups 3 and 4), and implant buccolingual resonance frequencies are shown in Fig. 10A (groups 1 and 2) and Fig. 10B (groups 3 and 4). As the distance between the implant apex and the sinus floor cortical bone lengthened (Fig. 9A), the values of the axial resonance frequencies increased significantly between models 1-1 and 1-2 in immediate and conventional loading. The resonance frequencies subsequently increased slowly. As the depth the implant apex penetrated into the sinus floor cortical bone increased, the axial resonance frequencies exhibited a linear upward tendency (Fig. 9B). The buccolingual resonance frequencies imperceptibly decreased as the distance between the implant apex and sinus floor cortical bone lengthened. No significant changes in frequency were observed when the penetration depth into the sinus floor cortical bone was adjusted (Fig. 10).

## Discussion

Mechanical analysis using the FE method has previously been utilized to reliably and accurately reveal the biomechanical behavior around dental implants without the risk or expense of implantation (2). The sinus area of the posterior maxilla is complex and it is not easy to establish an accurate and valid 3D FE model. In FEM research of a maxilla sinus area implant, Okumura *et al* (24) found no marked difference between conventional simplified 3D FE models and the full maxilla model created from computed tomography (CT) Digital Imaging and Communications in Medicine data. In order to exclude the influence of the anatomical variations of bone and to improve the comparability of the models, as suggested by Akca and Cehreli (25), the models used in the present study required a change in the height of the alveolar bone and the thickness of the sinus floor cortical bone. It was decided not to use an anatomical model of the maxilla provided by cone beam CT data; instead, 3D CAD models based on the anatomical data of the sinus area have been developed in this study.

Due to the poor quality and size of the alveolar ridge, the success rate of sinus area implantation is relatively low. Numerous studies have been conducted into dental implantation in the posterior maxilla (26,27), and clinical studies, animal experiments and FEM studies (28–30) have been carried out regarding the influence of bone quality and size on implants; however, to the best of our knowledge, no study concerning the association between an implant and the sinus floor cortical bone has been conducted. Sinus floor cortical bone has a tendency to be thin, which has made it less important in the research of dental implantation in the sinus area.

The results of the present study showed that the association between the implant apex and the sinus floor cortical bone affected the stress distribution of the cortical bone, the implant micromotion and the implant resonance frequencies. In the clinic, it is usual to select an implant that is a little shorter than the height of the alveolar ridge to keep the implantation safe. The study showed that if the height of the alveolar ridge is much longer than the implant length, it may not benefit the stability of the implant. This was particularly true when the implant apex made contact with or broke into or through the sinus floor cortical bone, when the maximum von Mises stress of the crestal cortical bone around the implant neck was reduced and whether loading was immediate or conventional. When the implant apex just broke through the sinus floor cortical bone in immediate loading (bicortical implantation), the sinus floor cortical bone suffered more stress than the implant neck cortical bone, which may increase the success rate of implantation. Although, the maximum von Mises stress of the sinus membrane was significantly increased in bicortical implantations, the sinus membrane was not supposed to be aggravated. Clinical and animal studies have shown that sinus membrane contact with the implant apex in sinus floor elevation without bone grafts also has a good success rate, without inflammation of aggravation of the sinus membrane (30,31).

With regard to implant micromotion in the condition of immediate loading, when the implant apex made contact with the lower surface of the sinus floor cortical bone or broke into the sinus floor cortical bone, implant displacements of the neck and apex decreased significantly. This result indicated that the sinus floor cortical bone was beneficial to the initial stability of the implant. In particular, when the implant apex was inside the sinus floor cortical bone, implant micromotion was reduced, and not too much stress passed to the sinus membrane; thus, a situation where the sinus floor cortical bone is thick enough to insert the implant apex inside it but without breaking through may be a better design of surgical treatment. For conventional loading, due to good osseointegration, the bone around the implant apex may not greatly affect the implant stability.

RFA as a nondestructive measurement has been widely used in clinical practice in the last five years (11) but only buccolingual resonance frequencies are checked. There are, in fact, numerous types and directions of resonance frequencies that cannot be examined, particularly the axial resonance frequency. In an FEA study, both buccolingual and axial resonance frequencies can be tested. The present results showed that, as the implant apex moved closer to the sinus floor cortical bone and within it, the implant axial resonance frequency increased but a change in the buccolingual resonance frequency was not evident. This suggests that the sinus floor cortical bone was beneficial in reducing implant axial resonance frequency, particularly when the implant apex was inside the sinus floor cortical bone. This means that the sinus floor cortical bone can improve implant stability in the axial direction but not the buccolingual direction.

In conclusion, this FE study of the association between the implant apex and the sinus floor cortical bone showed that the sinus floor cortical bone is beneficial for implant stability, particularly for immediate loading. In the situation where the implant apex contacts with, breaks into or breaks through the sinus floor cortical bone a significant reduction in the maximum von Mises stress of the sinus floor cortical bone, implant displacement and axial resonance frequencies can be observed. Further research concerning bicortical dental implantation in the posterior maxilla is required.

## Figures and Tables

**Table I:** Properties ascribed to materials used in the finite element models.

Material	Young’s modulus (MPa)	Poisson’s ratio	Mass density (g/cm^3^)
Titanium implant^a^	103,400	0.35	4.5
Cortical bone^a^	13,700	0.30	2.0
Cancellous bone (D3)^a^	1,370	0.30	1.0
Sinus membrane^b^	58	0.45	1.0

a Reference 19;

b references 20 and 21; D3, 3^rd^ class of the human alveolar bone classification according to Lekholm and Zarb (23).

## References

[1] Winter W, Krafft T, Steinmann P, Karl M (2011). Quality of alveolar bone - structure-dependent material properties and design of a novel measurement technique. J Mech Behav Biomed Mater.

[2] Ding X, Zhu XH, Liao SH, Zhang XH, Chen H (2009). Implant-bone interface stress distribution in immediately loaded implants of different diameters: a three-dimensional finite element analysis. J Prosthodont.

[3] Meredith N, Books K, Friberg B, Jemt T, Sennerby L (1997). Resonance frequency measurements of implant stability in vivo. A cross-sectional and longitudinal study of resonance frequency measurements on implants in the edentulous and partially dentate maxilla. Clin Oral Implants Res.

[4] Mohammed Ibrahim M, Thulasingam C, Nasser KS, Balaji V, Rajakumar M, Rupkumar P (2011). Evaluation of design parameters of dental implant shape, diameter and length on stress distribution: a finite element analysis. J Indian Prosthodont Soc.

[5] Lee JH, Frias V, Lee KW, Wright RF (2005). Effect of implant size and shape on implant success rates: a literature review. J Prosthet Dent.

[6] Toniollo MB, Macedo AP, Rodrigues RC, Ribeiro RF, de Mattos Mda G (2012). Three-dimensional finite element analysis of stress distribution on different bony ridges with different lengths of morse taper implants and prosthesis dimensions. J Craniofac Surg.

[7] Jeong CM, Caputo AA, Wylie RS, Son SC, Jeon YC (2003). Bicortically stabilized implant load transfer. Int J Oral Maxillofac Implants.

[8] Geng JP, Tan KB, Liu GR (2001). Application of finite element analysis in implant dentistry: a review of the literature. J Prosthet Dent.

[9] Van Staden RC, Guan H, Loo YC (2006). Application of the finite element method in dental implant research. Comput Methods Biomech Biomed Engin.

[10] Bozkaya D, Muftu S, Muftu A (2004). Evaluation of load transfer characteristics of five different implants in compact bone at different load levels by finite elements analysis. J Prosthet Dent.

[11] Chang PC, Lang NP, Giannobile WV (2010). Evaluation of functional dynamics during osseointegration and regeneration associated with oral implants. Clin Oral Implants Res.

[12] Huang HM, Lee SY, Yeh CY, Lin CT (2002). Resonance frequency assessment of dental implant stability with various bone qualities: a numerical approach. Clin Oral Implants Res.

[13] Pattijn V, Van Lierde C, Van der Perre G, Naert I, Vander Sloten J (2006). The resonance frequencies and mode shapes of dental implants: Rigid body behaviour versus bending behaviour. A numerical approach. J Biomech.

[14] Pommer B, Unger E, Sütö D, Hack N, Watzek G (2009). Mechanical properties of the Schneiderian membrane in vitro. Clin Oral Implants Res.

[15] Underwood AS (1910). An inquiry into the anatomy and pathology of the maxillary sinus. J Anat Physiol.

[16] Ulm CW, Solar P, Gsellmann B, Matejka M, Watzek G (1995). The edentulous maxillary alveolar process in the region of the maxillary sinus - a study of physical dimension. Int J Oral Maxillofac Surg.

[17] van den Bergh JP, ten Bruggenkate CM, Disch FJ, Tuinzing DB (2000). Anatomical aspects of sinus floor elevations. Clin Oral Implants Res.

[18] Gosau M, Rink D, Driemel O, Draenert FG (2009). Maxillary sinus anatomy: a cadaveric study with clinical implications. Anat Rec (Hoboken).

[19] Rues S, Lenz J, Schierle HP, Schindler HJ, Schweizerhof K (2004). Simulation of the sinus floor elevation. Proc Appl Math Mech.

[20] Huang CC, Chen LW, Wu DF, Chen YC (2012). Finite element simulations of the contact stress between rotary sinus lift kit and sinus membrane during lifting process. Life Sci J.

[21] Mellal A, Wiskott HW, Botsis J, Scherrer SS, Belser UC (2004). Stimulating effect of implant loading on surrounding bone. Comparison of three numerical models and validation by in vivo data. Clin Oral Implants Res.

[22] Morneburg TR, Pröschel PA (2002). Measurement of masticatory forces and implant loads: a methodologic clinical study. Int J Prosthodont.

[23] Lekholm U, Zarb GA, Albrektsson T (1985). Tissue Integrated Prostheses: Osseointegration in Clinical Dentistry.

[24] Okumura N, Stegaroiu R, Nishiyama H, Kurokawa K, Kitamura E, Hayashi T, Nomura S (2011). Finite element analysis of implant-embedded maxilla model from CT data: comparison with the conventional model. J Prosthodont Res.

[25] Akca K, Cehreli MC (2006). Biomechanical consequences of progressive marginal bone loss around oral implants: a finite element stress analysis. Med Biol Eng Comput.

[26] Cannizzaro G, Felice P (2013). Early implant loading in the atrophic posterior maxilla: 1-stage lateral versus crestal sinus lift and 8 mm hydroxyapatite-coated implants. A 5-year randomised controlled trial. Eur J Oral Implant.

[27] Doan N, Du Z, Crawford R, Reher P, Xiao Y (2012). Is flapless implant surgery a viable option in posterior maxilla? A review. Int J Oral Maxillofac Surg.

[28] Wang D, Künzel A, Golubovic V (2013). Accuracy of peri-implant bone thickness and validity of assessing bone augmentation material using cone beam computed tomography. Clin Oral Investig.

[29] Chou IC, Lee SY, Wu MC, Sun CW, Jiang CP (2014). Finite element modelling of implant designs and cortical bone thickness on stress distribution in maxillary type IV bone. Comput Methods Biomech Biomed Engin.

[30] Sul SH, Choi BH, Li J, Jeong SM, Xuan F (2008). Histologic changes in the maxillary sinus membrane after sinus membrane elevation and the simultaneous insertion of dental implants without the use of grafting materials. Oral Surg Oral Med Oral Pathol Oral Radiol Endod.

[31] Pjetursson BE, Rast C, Brägger U, Schmidlin K, Zwahlen M, Lang NP (2009). Maxillary sinus floor elevation using the (transalveolar) osteotome technique with or without grafting material. Part I: Implant survival and patients’ perception. Clin Oral Implants Res.

